# Connecting Grandparent Caregivers Through Telemental Health During COVID-19

**DOI:** 10.1093/geroni/igab046.1004

**Published:** 2021-12-17

**Authors:** Laura Bronstein, Kelley Cook, Youjung Lee

**Affiliations:** 1 Binghamton University, Binghamton University/Binghamton, New York, United States; 2 Community Research and action, Binghamton University/ Binghamton, New York, United States; 3 Binghamton University, Department of Social Work, Binghamton, New York, United States

## Abstract

Since the COVID-19 outbreak, children and their caregivers throughout the world are experiencing unprecedented long-term social isolation. For too many, especially grandparent-headed families, underrepresented minorities, and those living in poverty, this precipitates and exacerbates mental health conditions including anxiety and depression. Despite these families’ increased needs for mental health services during the pandemic, professionals often lack experience and expertise in telemental health, which is a safe and effective way to provide these services. In this symposium, we will present a telemental health model for working with grandparent-headed families that draws upon Solution-Focused Brief Therapy (SFBT), an evidence-based approach focusing on strengths. This SFBT-based telemental health training program prepares mental health professionals to implement this safe and innovative intervention, enabling them to effectively serve isolated and marginalized grandparent caregivers and their families when providing in-person services is not possible.

